# How low can you go: What is the safe threshold for platelet transfusions in patients with hematologic malignancy in sub-Saharan Africa

**DOI:** 10.1371/journal.pone.0211648

**Published:** 2019-02-06

**Authors:** Henry Ddungu, Elizabeth M. Krantz, Isaac Kajja, Sandra Naluzze, Hanifah Nabbanja, Flavia Nalubwama, Warren Phipps, Jackson Orem, Noah Kiwanuka, Anna Wald

**Affiliations:** 1 Uganda Cancer Institute, Upper Mulago Hill Road, Kampala, Uganda; 2 Vaccine and Infectious Disease Division, Fred Hutchinson Cancer Research Center, Seattle, WA, United States of America; 3 School of Medicine, College of Health Sciences, Makerere University Kampala, Uganda; 4 Department of Medicine, University of Washington, Seattle, WA, United States of America; 5 School of Public Health, College of Health Sciences, Makerere University Kampala, Uganda; 6 Department of Laboratory Medicine, University of Washington, Seattle, WA, United States of America; 7 Department of Epidemiology, University of Washington, Seattle, WA, United States of America; University of Witwatersrand/NHLS, SOUTH AFRICA

## Abstract

**Background:**

Despite the importance of platelet transfusions in treatment of hematologic cancer patients, the optimal platelet count threshold for prophylactic transfusion is unknown in sub-Saharan Africa.

**Methods:**

We followed patients admitted to the Uganda Cancer Institute with a hematological malignancy in 3 sequential 4-month time-periods using incrementally lower thresholds for prophylactic platelet transfusion: platelet counts ≤ 30 x 10^9^/L in period 1, ≤ 20 x 10^9^/L in period 2, and ≤ 10 x 10^9^/L in period 3. Clinically significant bleeding was defined as WHO grade ≥ 2 bleeding. We used generalized estimating equations (GEE) to compare the frequency of clinically significant bleeding and platelet transfusions by study period, adjusting for age, sex, cancer type, chemotherapy, baseline platelet count, and baseline hemoglobin.

**Results:**

Overall, 188 patients were enrolled. The median age was 22 years (range 1–80). Platelet transfusions were given to 42% of patients in period 1, 55% in period 2, and 45% in period 3. These transfusions occurred on 8% of days in period 1, 12% in period 2, and 8% in period 3. In adjusted models, period 3 had significantly fewer transfusions than period 1 (RR = 0.6, 95% CI 0.4–0.9; p = 0.01) and period 2 (RR = 0.5, 95% CI 0.4–0.7; p<0.001). Eighteen patients (30%) had clinically significant bleeding on at least one day in period 1, 23 (30%) in period 2, and 15 (23%) in period 3. Clinically significant bleeding occurred on 8% of patient-days in period 1, 9% in period 2, and 5% in period 3 (adjusted p = 0.41). Thirteen (21%) patients died in period 1, 15 (22%) in period 2, and 11 (19%) in period 3 (adjusted p = 0.96).

**Conclusion:**

Lowering the threshold for platelet transfusion led to fewer transfusions and did not change the incidence of clinically significant bleeding or mortality, suggesting that a threshold of 10 x 10^9^/L platelets, used in resource-rich countries, may be implemented as a safe level for transfusions in sub-Saharan Africa.

## Introduction

By the year 2030, it is estimated that there will be 1.28 million new cancer cases, 970,000 cancer deaths, and a near doubling of leukemia and lymphoma cases in sub-Saharan Africa (SSA) [1]. Hematologic malignancies account for nearly 10% of the overall cancer burden in SSA [2]. Such patients often develop severe thrombocytopenia resulting in risk of bleeding [3–5]. Platelet transfusions can be given to prevent or stop bleeding [6, 7]. A 1962 study by Gaydose, L. A., and others [3], is usually cited as justification for administering prophylactic platelet transfusion at a platelet count of ≤ 20 x10^9^/L. More recently, a lower threshold for platelet transfusion has been advocated, based on studies in patients with leukemia and hematopoietic stem cell transplants and the current international guidelines recommend a transfusion trigger of 10x10^9^/L or lower (5 x 10^9^/L) for stable patients without additional risk factors [8–11].

Administration of platelets can be associated with significant toxicity or transmission of infections. Both the benefits and risks for platelet transfusion may differ in resource-rich and resource-poor countries. The optimal platelet count threshold for prophylactic transfusion in SSA is not known, and the currently available international guidelines may be less relevant in this setting.

The aim of the study was to develop and implement local guidelines for platelet transfusions in Ugandan cancer patients with thrombocytopenia.

## Materials and methods

### Study participants and data collection

We conducted a prospective study among patients admitted to the Uganda Cancer Institute (UCI) with a hematological malignancy from October 2014 to October 2015. The UCI is a multidisciplinary comprehensive cancer center located within the Mulago Hospital Complex that was established in 1967 with an objective to carry out cancer research, training, and clinical care for cancer patients in Uganda [12]. The institute maintains an inpatient facility of 100 beds and registers on average, 30 new patients with a diagnosis of a hematological malignancy, excluding Burkitt lymphoma, per month. The Uganda Blood Transfusion Service (UBTS) is a government agency responsible for providing transfusion services to all regions of Uganda. It has a mandate to provide sufficient and safe blood based on voluntary non-remunerated blood donation as endorsed by the World Health Organization (WHO) in accordance with World Health Assembly resolution 28.72, adopted in 1975 [13]. UCI receives all its blood products, including platelets from UBTS.

With a primary objective of comparing the proportion of patients who develop WHO grade ≥ 2 bleeding when prophylactic platelet transfusions are based on three different transfusion thresholds, we implemented local guidelines for platelet transfusion among Ugandan cancer patients with thrombocytopenia through a sequential study approach. All in-patients with a hematological malignancy during the study period were included and managed according to current practice (usual care) at the UCI. To determine a safe threshold for prophylactic platelet transfusions, different platelet count thresholds (≤ 30 x 10^9^/L, ≤ 20 x 10^9^/L and ≤ 10 x 10^9^/L) were specified for a 4-month period each. These proposed levels were discussed with the clinicians and they agreed to adhere to these threshold levels for the specified time periods. At the end of each 4-month period the provisional observations were shared with the clinicians and the threshold was lowered to the next level. There was no blinding of either clinicians or patients and the treating clinician’s decision was accepted.

Standardized forms were used to extract demographic and clinical data, including blood counts, transfusions given, and presence of bleeding, from patient medical records. Blood counts were normally measured twice a week, but could be ordered more frequently, at the doctor’s request. Presence of bleeding was based on notes in the patient chart; if the patient was seen by the doctor but there was no mention of bleeding in the chart notes, we assumed the patient had no bleeding on that day. Bleeding status was treated as missing (not assessed) on days where the patient was not seen by the doctor or there were no chart notes. Because bleeding was a primary outcome, we excluded participants who had no days with bleeding assessed during the study. We assumed that all transfusions were recorded in patient charts and so days without mention of transfusions were considered to have no transfusions.

The number of platelet units that UCI requested and received during the study period was abstracted from the UBTS records. A platelet unit refers to 60-80ml whole blood–derived platelet concentrates. Participants were followed until they were discharged or died, for up to 30 days.

The study was approved by the Makerere University School of Medicine Research and Ethics Committee, the Fred Hutchinson Cancer Research Center (FHCRC) IRB and the Uganda National Council for Science and Technology. All participants signed an informed consent form.

### Statistical analyses

Clinically significant bleeding was defined as WHO grade ≥ 2 bleeding and a platelet transfusion was defined as having at least one 60-80ml whole blood-derived platelet unit. We compared the frequency of clinically significant bleeding and platelet transfusions by study period using generalized estimating equations (GEE) with Poisson distribution and log link to account for correlation among longitudinal binary outcomes measured in the same participant [14]. Model estimates for study period comparisons were presented as relative risks (RR) with 95% confidence intervals (CI). Platelet counts during follow-up were log_10_-transformed for comparisons among study periods which used GEE with the normal distribution.

We computed the proportion of patients that died in each study period, and compared this in-patient mortality using Poisson regression with robust standard errors. For all models, we computed both unadjusted estimates and estimates adjusted for the following baseline factors: age, sex, cancer type, chemotherapy use, platelet count and hemoglobin level. Adherence to the platelet count threshold for transfusion in each study period was defined using two different components. First, we considered all days with the platelet count under the trigger threshold and computed the proportion of those days with a platelet transfusion given within 1 day of the low platelet count. Next, we considered all days with platelet counts above the trigger threshold and computed the proportion of those days where platelet transfusions were (appropriately) not given for prophylaxis reasons.

## Results

### Cohort characteristics

We consented 80, 83 and 74 patients for study periods 1, 2 and 3, respectively. Two patients from period 2 were excluded from the analysis due to lack of hematological malignancy and 18, 12, and 17 patients from periods 1, 2, and 3, respectively, were excluded because they had no days with bleeding assessed. The total number of patients enrolled in each of the 3-time periods and eligible for analysis was 62, 69 and 57, with 998, 1300, and 1047 follow up days, respectively. Enrolled patients were followed for a median of 13 days (range 2–31 days) in study period 1, 19 days (range 2–30) period 2, and 17 days (range 3–31) in period 3. Of the 62 patients enrolled in period 1, eight had follow-up data continue into period 2 and of the 69 patients enrolled in period 2, eight had follow-up data continue into period 3.

Baseline participant characteristics are shown in Table 1. The participants originated from various geographical regions in Uganda. For the three time periods, 140 (74.5%) participants were HIV negative, 8 (4.3%) HIV positive and 40 (21.3%) had an unknown HIV status. Acute lymphoblastic leukemia (ALL), acute myeloid leukemia (AML), and non-Hodgkin lymphoma (NHL) accounted for most malignancies. ALL was more common in study period 1 than in study periods 2 and 3, while AML was less common in period 1 than in periods 2 and 3. The median baseline platelet count was slightly higher in period 1 than in period 2 and 3, though each period had a wide range of baseline platelet counts.

**Table 1:** Baseline characteristics of participants enrolled in each of the three-time periods^1^.

Characteristic	Period 1 (n = 62)	Period 2 (n = 69)	Period 3 (n = 57)	Total (n = 188)
**Demographics:**				
Age, median (range)	18 (1–78)	26 (1–80)	22 (2–75)	22 (1–80)
Male: Female, n (%)	40:22 (64.5:35.5%)	39:30 (56.5:43.5%)	36:21 (63.2:36.8%)	115:73 (61.2:38.8%)
**Nationality**, n (%)				
Uganda	62 (100%)	66 (95.7%)	57 (100%)	185 (98.4%)
Kenya	0	1 (1.5%)	0	1 (0.5%)
Rwanda	0	1 (1.5%)	0	1 (0.5%)
Other	0	1 (1.5%)	0	1 (0.5%)
**Clinical Characteristics:**				
**Medication (current or past), n**^**2**^ **(%)**:				
Chemotherapy	28 (45.2%)	27 (39.1%)	22 (38.6%)	77 (41.0%)
NSAIDs	1 (1.6%)	2 (2.9%)	0	3 (1.6%)
**Primary Diagnosis**, **n (%)**				
Acute Leukemia				
Acute lymphocytic leukemia	26 (41.9%)	20 (29.0%)	14 (25.5%)	60 (32.3%)
Acute myeloid leukemia	6 (9.7%)	19 (27.5%)	14 (25.5%)	39 (21.0%)
Chronic Leukemia				
Chronic myeloid leukemia	5 (8.1%)	8 (11.6%)	4 (7.3%)	17 (9.1%)
Chronic lymphocytic leukemia	0	5 (7.3%)	6 (10.9%)	11 (5.9%)
Lymphoma				
Hodgkin’s lymphoma	6 (9.7%)	5 (7.3%)	6 (10.9%)	17 (9.1%)
Non-Hodgkin lymphoma	11 (17.7%)	7 (10.1%)	6 (10.9%)	24 (12.9%)
Primary CNS lymphoma	0	0	1 (1.8)	1 (0.5%)
Others				
Multiple myeloma	7 (11.3%)	5 (7.3%)	4 (7.3%)	16 (8.6%)
Myelodysplastic syndrome	1 (1.6%)	0	0	1 (0.5%)
**Baseline laboratory studies,** median (range)				
Hemoglobin (g/dL)	7.4 (2.2–14.8)	7.4 (2.6–13.7)	7.0 (3.4–16.0)	7.4 (2.2–16)
Platelet count (x10^9^/L)	41.5 (3.0–667.0)	34.0 (1.0–674.0)	33.0 (2.0–956.0)	37 (1–956)
WBC (x10^9^/L)	8.5 (0.2–551.0)	7.6 (0.2–647.8)	11.0 (1.0–679.6)	9.0 (0.2–679.6)

^1^Baseline defined as at study enrollment

^2^n, number of patients

### Platelet counts and platelet transfusions

**Fig 1:**
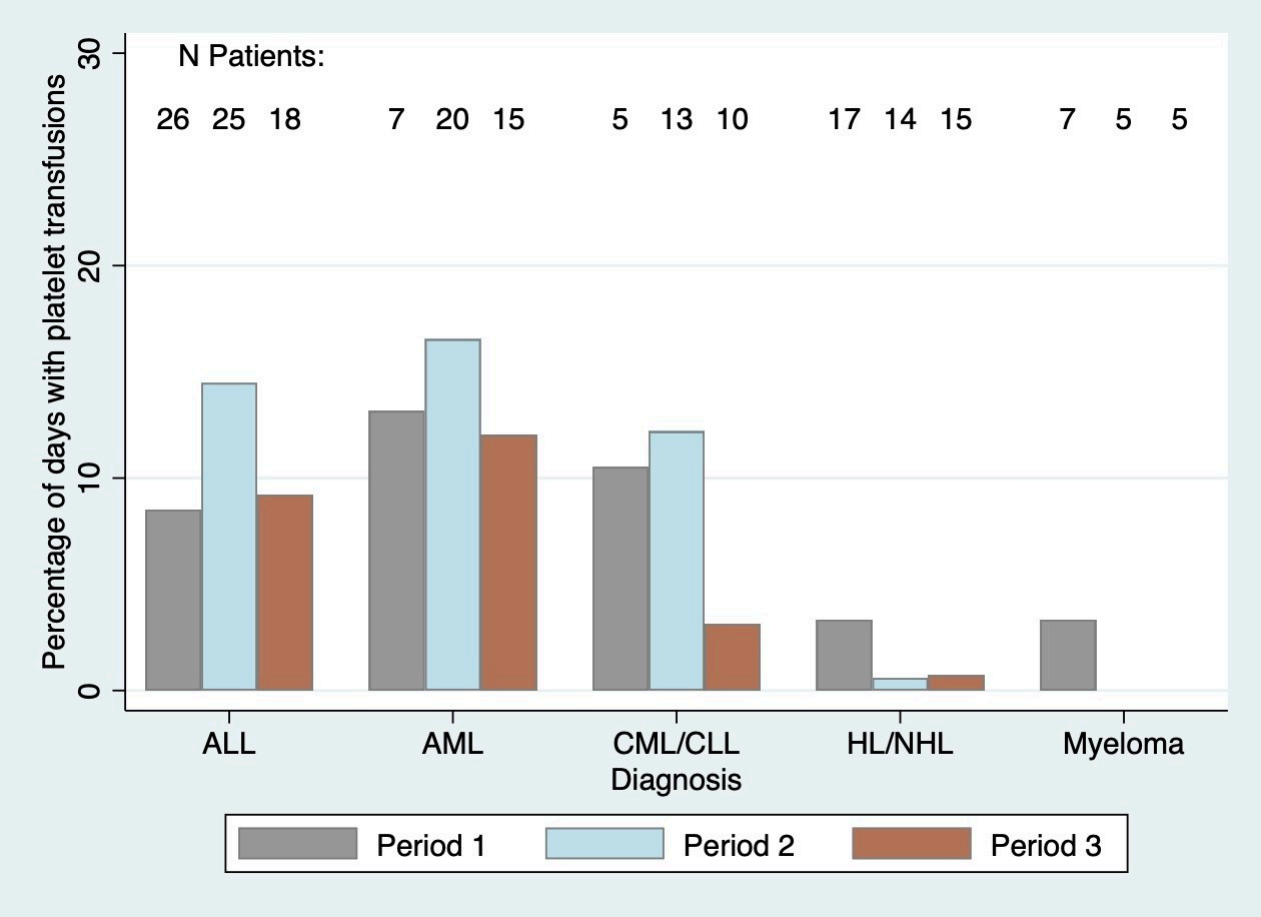
Percentage of days with platelet transfusion by both time period and cancer type.

Summaries of platelet counts and administered platelet transfusions during follow up for each of the 3 time periods are shown in Table 2. There was no significant difference in the mean platelet counts between the 3-time periods in both unadjusted (p = 0.14) and adjusted (p = 0.55) models. Platelet transfusions were more common among patients with ALL, AML, and CML or CLL than among patients with lymphomas or myeloma (Fig 1). In unadjusted analysis, platelet transfusions were more frequent in period 2 compared to both period 1 (RR = 1.6, 95% CI 1.0–2.5; p = 0.03) and period 3 (RR = 1.5, 95% CI 1.0–2.3; p = 0.04). In models adjusted for age, sex, cancer type, chemotherapy use, baseline platelet count and baseline hemoglobin, period 2 no longer had significantly greater frequency of platelet transfusions relative to period 1 (RR = 1.2, 95% CI 0.9–1.7; p = 0.27), and period 3 had significantly fewer transfusions compared to both period 1 (RR = 0.6, 95% CI 0.4–0.9; p = 0.01) and period 2 (RR = 0.5, 95% CI 0.4–0.7; p<0.001). Most platelet transfusions were given as prophylaxis for low platelets. Corresponding to the decreasing thresholds recommended for platelet transfusion during the study, the median pre-transfusion platelet counts were 10, 7 and 5 x10^9^/L, for the 3-time periods, respectively, though the ranges were wide.

**Table 2:** Distribution of platelet counts and frequency of platelet transfusions among patients with hematological malignancies during the three study periods.

Platelet count results and administered transfusion therapy	Period 1 (n = 62)	Period 2 (n = 77)	Period 3 (n = 65)
**Platelet Counts**			
Median number of days per patient with platelets measured (range)	3 (1–11)	4 (1–11)	5 (1–10)
Platelet count, number of days (%):			
≤ 10 x 10^9^ / L	44 (18.8%)	98 (26.5%)	123 (37.7%)
> 10 x 10^9^ / L to ≤ 20 x 10^9^ / L	38 (16.2%)	82 (22.2%)	53 (16.3%)
> 20 x 10^9^ / L to ≤ 30 x 10^9^ / L	21 (9.0%)	29 (7.8%)	22 (6.8%)
> 30 x 10^9^ / L to ≤ 50 x 10^9^ / L	32 (13.7%)	40 (10.8%)	27 (8.3%)
> 50 x 10^9^ / L	99 (42.3%)	121 (32.7%)	101 (31.0%)
**Platelet transfusion**			
Number of patients with at least one platelet transfusion (%)	26 (41.9%)	42 (54.6%)	29 (44.6%)
Number of days with platelet transfusion / Days assessed (%)	62/842 (7.4%)	151/1268 (11.9%)	83/1058 (7.8%)
Reason for transfusion, n (%):			
Prophylactic for low platelets	37 (59.7%)	82 (54.3%)	45 (54.9%)
Therapeutic for active bleeding	17 (27.4%)	48 (31.8%)	21 (25.6%)
Pre-invasive procedure	0	0	0
Other^1^	8 (12.9%)	21 (13.9%)	16 (19.5%)
Median pre-transfusion platelet count (x10^9^/L); (range)	10 (0–62)	7 (0–73)	5 (1–35)
Number of platelet units given per platelet transfusion^2^ (%)			
1	16 (29.6%)	31 (20.8%)	30 (36.1%)
2	30 (55.5%)	90 (60.4%)	44 (53.0%)
3	7 (13.0%)	20 (13.4%)	9 (10.8%)
≥4	1 (1.9%)	8 (5.4%)	0 (0.0%)

^1^Other indicates that the reason for transfusion was not documented or specific reasons given were other than those shown in the table, e.g. “bleeding, but not acute bleeding”.

^2^On days with more than one transfusion, the number of units shown are the total for the entire day. Number of platelet units was unknown for 8 transfusions in period 1 and 2 in period 2.

The total number of 60-80mL whole blood-derived platelet concentrate units requested and received for the entire UCI was available from UBTS records for 289 days of the 12-month study period. During this time, the mean number of 60-80mL platelet concentrate units requested for the entire UCI per day was 17 for period 1; 21 for period 2; and 25 for period 3 but the mean platelet units received was only 5.8, 6.4 and 7.7, respectively. These corresponded to a median percentage of requested platelet units received per day of 36.3% (range 0–110%), 32.3% (range 0–110%), and 30% (range 0–100%). The total number of platelet units transfused to study participants during the 3 study periods was 101, 304 and 145, respectively. Most transfusions had 2 platelet units given with only a very small proportion of transfusions receiving 4–6 units (the ideal for an adult patient) per transfusion (Table 2).

### Adherence to platelet transfusion thresholds

We first computed how often platelet transfusions were given when platelet counts fell below our study transfusion thresholds defined for each study period. Among all days with platelet counts below the trigger threshold, platelet transfusions were given within 1 day of the low platelet counts for 29 (32.2%) days in period 1, 76 (45.8%) days in period 2, and 40 (33.9%) in period 3. Next, we computed how often platelet transfusions for prophylaxis were appropriately avoided when platelet counts were above study transfusion thresholds. Among such days with platelet counts above the trigger threshold, no platelet transfusions for prophylaxis were given on nearly all days in all 3 study periods (103 days (99.0%) for period 1, 165 (97.6%) for period 2, and 184 (99.5%) for period 3). Had the trigger threshold not been lowered for periods 2 and 3, we estimated that an additional 26 platelet transfusions would have been performed for prophylaxis in periods 2 and 3.

### Bleeding and mortality

**Fig 2:**
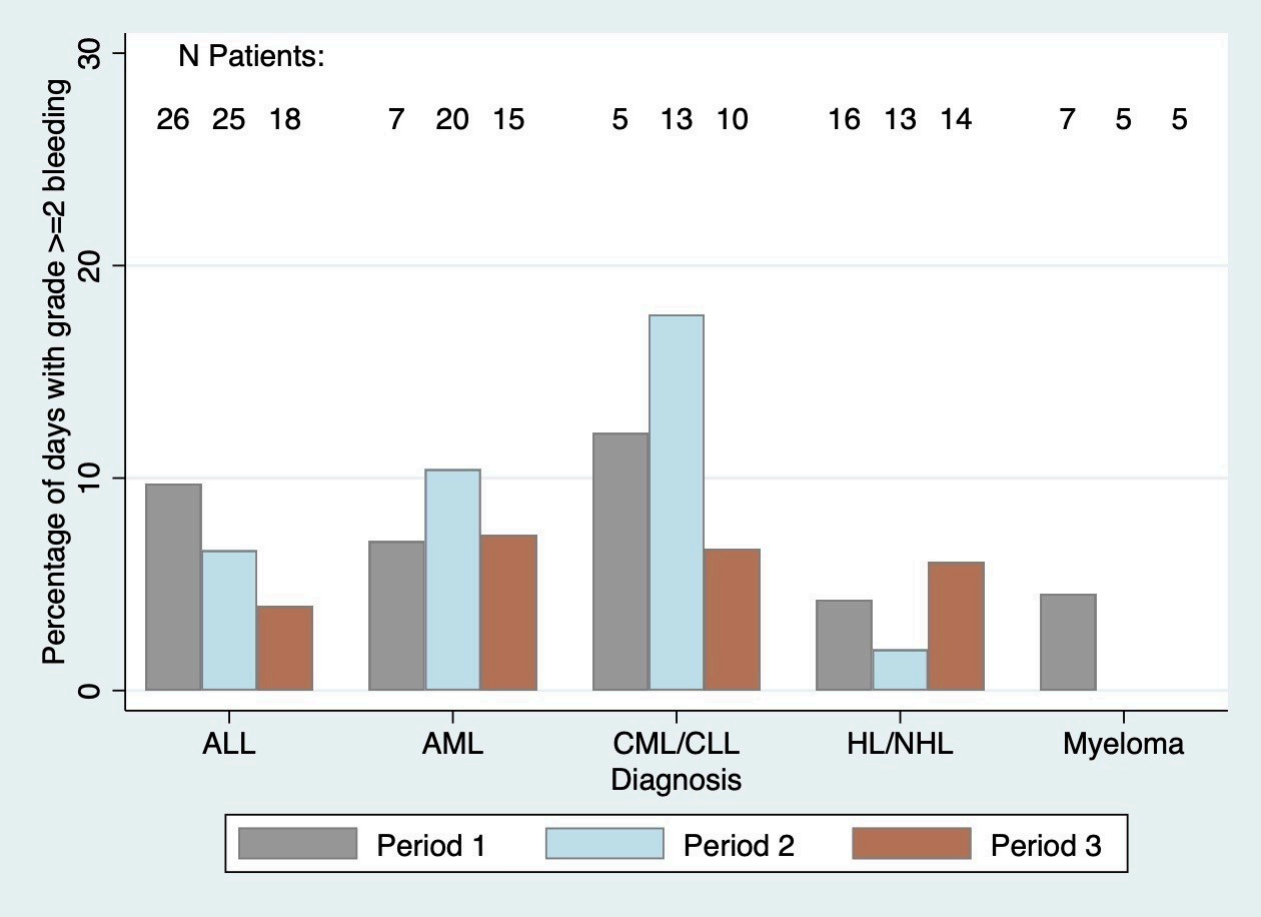
Percentage of days with grade 2 or higher bleeding by diagnosis in each of the time periods.

Bleeding was assessed on a median of 6 days per patient (range 1–30 days) in period 1, 9 days (range 1–29) in period 2, and 10 days (range 1–29) in period 3. Eighteen patients (29.5%), 23 (30.3%) and 15 (23.4%) had clinically significant bleeding on at least one day in period 1, 2 and 3, respectively. Only one patient had grade 3 bleeding, and this was for a single day in period 1; no patients had any grade 4 bleeding. The majority of the remaining patients with at least one day of bleeding had a maximum grade of grade 2 bleeding in each study period. Clinically significant bleeding was uncommon among all days with bleeding assessed for all 3 study periods and was especially rare among patients with lymphomas or myeloma (Fig 2). Of the 559 total days with bleeding assessed in time period 1, 45 days (8.1%) had clinically significant bleeding, 72 of 809 (8.9%) days had clinically significant bleeding in period 2 and 38 of 755 (5.0%) in period 3. As noted above, platelet transfusions were administered on only 32–46% of days with platelet counts below recommended threshold. There was no significant difference in the frequency of clinically significant bleeding by study period in both unadjusted models (p = 0.30) and adjusted models (p = 0.41).

Thirteen (21.0%) patients died in period 1, 15 (21.7%) in period 2, and 11 (19.3%) in period 3 (adjusted p = 0.96). Among those who died, only one patient in period 2 and one in period 3 had bleeding as the likely cause of their death.

## Discussion

In a study introducing sequentially lower thresholds for platelet transfusion for patients with hematologic malignancies at the UCI, we found no significant difference in the frequency of clinically significant bleeding or inpatient mortality when prophylactic platelet transfusions were given at ≤30 x 10^9^/L, ≤20 x 10^9^/L, or ≤10 x 10^9^/L in both unadjusted and adjusted models. Importantly, in models adjusted for potential confounders, the number of transfusions provided was substantially lower in the later time periods. To our knowledge, this is the only study that has locally generated evidence to inform guidelines for a safe threshold for prophylactic platelet transfusion in SSA.

Thrombocytopenia is a well-recognized complication of hematological malignancies due to the disease and/or high dose chemotherapy used in the treatment of such diseases. Allogeneic prophylactic platelet transfusion is widely used to reduce the risk of bleeding. The AABB (formerly, the American Association of Blood Banks) recommends that hospitalized adult patients with treatment-induced thrombocytopenia should be transfused prophylactically to reduce the risk of spontaneous bleeding [11]. However, platelet transfusions are not without risk; they may be associated with morbidity and mortality, and therefore should only be administered when needed. Several studies and clinical practice guidelines have recommended a lower prophylactic platelet count trigger of 10 x 10^9^/L [8, 10, 11, 15] as morbidity due to spontaneous bleeding is rare with higher counts. Studies to compare the frequency and severity of bleeding in patients who received prophylactic platelet transfusions under two different protocols, found it safe to decrease the prophylactic-transfusion threshold from 20x10^9^/L to 10x10^9^/L in adults with leukemia with no significant effect on morbidity [16, 17]. A prospective clinical trial of prophylactic platelet transfusion and bleeding incidence in hematopoietic stem cell transplant patients also found that a platelet transfusion trigger of 10 x 10^9^/L was safe [18]. Another study that compared a trigger of less than 10 x 10^9^/L with that of less than 30 x 10^9^/L concluded the safety of < 10 x 10^9^/L resulting in a decreased use of platelets [19]. This therefore implies that a lower platelet count threshold was not associated with more bleeding, similar to what other studies have described [20, 21] and supports moving away from the ≤20 × 10^9^/L or ≤30 × 10^9^/L threshold.

Higher platelet counts may be appropriate for patients who are undergoing invasive procedures such as lumbar puncture. At our institution, a platelet transfusion is given to thrombocytopenic patients mostly when patients are bleeding but also prophylactically (if available) when platelet counts are less than 10 x 10^9/L and no additional comorbidities are present, or at the discretion of the attending physician. Data on the recommended threshold for platelet transfusions in thrombocytopenic patients with leukemia undergoing lumbar puncture is limited. A study to assess the safety of lumbar puncture for patients with acute leukemia with restrictions on prophylactic platelet transfusions documented an increased occurrence of procedure related traumatic bleeding which could result in serious consequences. The authors therefore recommended a trigger of 20 x 10^9/L and less for prophylactic platelet transfusion prior to performing a lumbar puncture [22]. However, an earlier study recommended a prophylactic platelet count of 40 x 10^9^/L as a safe count for lumbar puncture [23], and the AABB suggests prophylactic platelet transfusion for patients having elective diagnostic lumbar puncture with a platelet count less than 50 x 10^9^/L [11].

In the current study, platelet transfusions were given on only 40% of the days with platelet counts below the study determined threshold for prophylactic platelet transfusions. This low rate of transfusion could be explained by the inadequate availability of platelets: the UCI received a mean of less than 8 units per day of 60-80mL platelet concentrate units from the blood bank. Uganda, like most other countries in SSA, does not have an adequate blood supply. SSA collects less than one-tenth of the world’s total blood supply fulfilling only 20–50% of its blood requirements. Factors including the transfusion medicine infrastructure, and transfusion policies such as voluntary non-remunerated blood donation (VNRBD) recommended by World Health Assembly resolution WHA63.12, in which blood, plasma or cellular components are given of the person’s own free will without receiving payment in cash, or any substitute for money [24], are hurdles to achieving a safe and adequate blood supply for SSA [25, 26].

Clinicians in our study adhered closely to the set guidelines by avoiding prophylactic platelet transfusions on nearly all days with platelet counts above the trigger threshold. This is similar to a New Zealand study that demonstrated a high rate of adherence to local transfusion policy in patients with hypo-proliferative thrombocytopenia and recommended educating staff in the use of a stringent transfusion policy to reduce unnecessary platelet transfusions [27]. A secondary analysis of the prophylactic platelet dose (PLADO) trial to determine whether bleeding outcomes might vary with age, assessed compliance with prophylactic transfusion at a trigger of 10 x 10^9^/L and found that physicians adhered on 92% of patient-days on study [28] which is similar to our study. However, contrary to our findings, a study in South Africa, to establish whether clinicians adhered to local platelet transfusion guidelines, showed poor compliance with local guidelines, with 34% of platelet transfusions not aligned with guidelines [29]. A UK national audit of the use of platelet transfusions also showed poor compliance with local guidelines [30]. Adherence to restrictive transfusion strategies may reduce the morbidity associated with blood product usage by avoiding unnecessary transfusions hence improved patient safety and reduce hospital costs [31]. Our provision of specific training and written guidelines for platelet transfusions are likely to have improved the adherence, as noted by others [29, 30, 32].

We acknowledge several limitations to this study. First, in this observational study, patients were not randomized to the three different platelet transfusion thresholds and, therefore, patients from each study period differed with respect to risk factors for our outcomes of bleeding and mortality. However, we adjusted for age, sex, cancer type, chemotherapy use, baseline platelet count and hemoglobin level in models comparing our outcomes by study period to minimize these differences. A randomized clinical trial was not feasible with the available resources, so we opted for this quasi-experimental design. Next, our data regarding bleeding relied on clinician notes recorded in patient charts during routine clinical care. Nearly 20% of our cohort had no chart notes available for the duration of the study and were excluded from analysis. It is unclear how these patients may have influenced our findings; inpatient follow-up tended to be relatively short for this group (median of 6 days) and the majority (81%) exited the study due to patient discharge, suggesting they may have been a healthier group. We may have missed some bleeding events among patients included in the analysis, though we feel it would be unlikely that episodes of severe bleeding would have been omitted from the patient chart. Our results were found in a transfusion medicine environment where platelet doses administered were substantially lower than those administered in resource rich environments; although this may be viewed as a limitation of our study it reflects the reality of blood supply that is available in resource restricted countries. We also note that certain malignancies such as ALL are overrepresented in the cohort while others such as MDS are very underrepresented. In addition, we were not able to divide patients into high- and low-grade types of lymphomas, nor account for underlying comorbidities. Finally, we did not capture whether there were instances when clinicians intended to perform a transfusion but could not due to shortages in platelet units. However, we obtained the total number of units requested and those received by UCI for the majority of study days to make inferences regarding the impact of platelet availability on platelet transfusions.

## Conclusion

Lowering the threshold for platelet transfusion among UCI inpatients with hematological malignancies led to fewer transfusions and did not change the incidence of clinically significant bleeding or mortality, suggesting that a threshold of 10 x 10^9^/L platelets, as used in resource-rich countries, may be a safe level for transfusions in SSA. Low availability of blood products hindered clinicians’ ability to provide transfusions when platelet counts dropped below our defined trigger thresholds. When platelet counts were above the thresholds, adherence to prophylactic platelet count thresholds was excellent, suggesting that clinicians were comfortable with the lower threshold.
